# Considerations for Remote Data Collection Among Adolescents During the COVID-19 Pandemic

**DOI:** 10.1016/j.jadohealth.2020.11.020

**Published:** 2021-03

**Authors:** Kara Hunersen, Astha Ramaiya, Chunyan Yu, Jakevia Green, Anggriyani Wahyu Pinandari, Robert Blum

**Affiliations:** a Department of Population and Family Health, Johns Hopkins University Bloomberg School of Public Health, Baltimore, Maryland; b Department of Epidemiology & Social Science, NHC Key Laboratory of Reproductive Regulation (Shanghai Institute of Planned Parenthood Research), Fudan University, Shanghai, China; c Institute of Women and Ethnic Studies, UNO Research and Technology Foundation, Inc, New Orleans, Louisiana; d Center for Reproductive Health, University Gadjah Mada, Daerah Istimewa Yogyakarta, Indonesia

The COVID-19 pandemic has forced a global shift in nonclinical research methodologies. Many studies have halted data collection to respect international restrictions on in-person contact. However, the demand for information has never been higher. This is especially true for children and adolescents who, in most countries of the world, have been out of the classroom and away from peers for months. Multiple studies from before the pandemic show the importance of peer interaction in child development [[1], [2], [3]], but what is the human cost of the pandemic for our younger citizens? This question validates the necessity for research among young people to fully understand the impacts of the current emergency. Although the pandemic created a need, it has also created a unique opportunity. The ubiquity of technology allows for remote data collection through the use of Web platforms, applications, and email [4]. However, studies of these modalities show that in-person data collection has higher response rates and better quality of data than remote data collection [5]. Thus, new remote data collection strategies are needed.

The Global Early Adolescence Study (GEAS) is a longitudinal study that follows the experiences of more than 15,000 adolescent boys and girls aged 10–14 years on five continents [6]. With pre-COVID data in multiple sites, the GEAS was in a unique position to use this as a baseline to assess how COVID-19 has affected the physical and mental health of young adolescents. Together with global partners, we developed a module to assess adolescents' knowledge of COVID-19, their experiences with social distancing, and how the pandemic has affected daily life. Before school closures, GEAS data collection was done primarily via computer-assisted self-interviewing using tablets in classrooms. This allowed anonymity while still ensuring data quality with interviewers available to answer questions or address issues [7]. Once schools were closed due to the pandemic, GEAS collaborators began contemplating how to shift to remote data collection with anonymity and without sacrificing quality.

Three sites have now implemented the COVID module with different strategies for remote data collection. Studies were approved by local and Johns Hopkins University institutional review boards (Ref #8549). The team in Shanghai, China, had a small number of students test an online version of the COVID module using phone or laptops, but then with the reopening of schools reverted to in-person surveys. In both Semarang and Denpasar, Indonesia, young people completed a fully online survey. In New Orleans, a fully online COVID module began in August 2020. What has been learned from these three experiences?

## Recruitment

Field teams were able to use contact information collected during the baseline of the GEAS to identify participants for the COVID module. In addition, because the longitudinal GEAS survey is conducted primarily in schools, teachers have an important role in the recruitment process. Both Indonesia and China teams capitalized on this relationship to facilitate remote recruitment. Teachers sent information about the survey to students, so they were prepared to hear from data collectors. In Semarang, teachers used WhatsApp groups to reach students and parents for both recruitment and data collection. Without parent–teacher engagement or prior knowledge of the survey in Denpasar, many parents thought the recruitment messages were spam and did not respond. This necessitated multiple text and phone follow-ups by the research team.

In New Orleans, the study team used a Web-based platform, *TextIt,* to send a technology access screener to students using a local telephone number that would not be recognized as spam. This circumvented emails and individual phone calls and allowed young people to indicate their preferred mode of survey participation.

## Data Collection

Data collectors in China who tested the online forms before implementation reported issues with load time when using a phone, but not when using a computer, suggesting that Internet connection was not the issue. Thus, researchers encouraged adolescents to use computers when available. However, they also sent a notice to adolescents advising them of the longer load time with cell phones to decrease frustration and encourage them not to abandon the survey. The survey completion rate was 90.2%.

Both sites in Indonesia had difficulties with data collection, often related to communication challenges between the study team and participants. An issue that emerged early on was with the WhatsApp group used by teachers and students to communicate about the study. When teachers were asked to send customized survey links to individual students, they instead sent the entire list of links to the group. As Internet needs are higher than before the pandemic, non-GEAS students were thus motivated to incorrectly fill out the survey to receive the data package incentive offered. In addition, participants did not communicate problems to the study team when they arose, such as limited data capacity. Without this knowledge, the study team was unable to purchase data packages for users until multiple rounds of follow-up eventually identified the issue. Finally, low signal quality caused a delay in survey data being uploaded to the main server, which made monitoring data quality difficult. The data managers had to check the server multiple times a day to ensure all the surveys were uploaded and from the correct participants.

New Orleans did not experience technical issues, although their survey completion rate was <25%. The technology access screener did indicate that several participants did not have consistent access to the Internet or devices. The study team speculates other potential issues include concern about survey length, lack of GEAS recognition 2 years after baseline, and fatigue with online school assignments similar to this survey. Strategies to address these issues are currently being explored.

## Lessons Learned

Through the experiences surveying young adolescents during the COVID-19 pandemic in three diverse contexts, we conclude (1) there is a central role for a known human interface; thus, local partners should encourage teachers to communicate with parents and young people; (2) study teams must assess the technological capacity and preferences of their target population; This will allow a smoother transition to remote data collection if adolescents are using platforms with which they are already familiar; (3) to enhance response rates in low-income settings, sufficient funds are needed to be able to offer free data and/or phone plans to facilitate adolescents' connectivity to participate; (4) data collectors must be well prepared to detect and correct potential issues and provide consistent follow-up with participants throughout the study period so that any new issues can be addressed and adjustments to the protocol made.
